# Clinical Characteristics and Outcomes of Patients Hospitalized with COVID-19 at Case Hospital, Uganda

**DOI:** 10.1155/2022/5477790

**Published:** 2022-06-08

**Authors:** Mirriam Apiyo, Ronald Olum, Amina Kabuye, Betty Khainza, Anne M. Amate, Vittal Byabashaija, Derrick Nomujuni, Kato Sebbaale, Peter Senfuka, Simon Kazibwe, Gurav Sharma, Lindsay Davidson, Felix Bongomin

**Affiliations:** ^1^Case Hospital, Kampala, Uganda; ^2^Department of Internal Medicine, St. Francis Hospital Nsambya, Kampala, Uganda; ^3^Department of Medical Microbiology & Immunology, Faculty of Medicine, Gulu University, Gulu, Uganda

## Abstract

Data on clinical outcomes of patients hospitalized with coronavirus disease 2019 (COVID-19) in private health facilities in Uganda is scarce. We conducted a retrospective cohort study of patients hospitalized with COVID-19 at Case Hospital, Kampala, Uganda, between June 2020 and September 2021. Data of 160 participants (median age 45 years (interquartile range [IQR]: 37–57) and 63.5% male) was analyzed. Seventy-seven (48.1%) participants had non-severe, 18 (11.3%) severe, and 83 (51.9%) critical COVID-19 illness. In 62 participants with chest computed tomography findings, 54 (87%) had bilateral disease, with 22 (35%) having ground-glass opacities. The median duration of hospitalization was 5 days (IQR: 3–9 days). Overall, 18 (11.3%) participants died. Survival at 14 and 28 days was 89% and 72%, respectively. Factors strongly associated with all-cause mortality were as follows: age >50 years (odds ratio [OR]: 8.6, 95% confidence interval [CI]: 1.1–69.2, and *p*=0.042), having at least 1 comorbidity (OR: 3.2, 95% CI: 1.1–8.9, and *p*=0.029), hypertension (OR: 3.2, 95% CI: 1.2–8.6, and *p*=0.024), diabetes mellitus (OR: 2.9, 95% CI: 1.0–8.5, and*p*=0.056), and oxygen saturation <92% (OR: 5.1, 95% CI: 1.8–14.4, and *p*=0.002). In this private health facility, mortality was about 1 in 10 patients, and more people presented with critical illness in the second wave of the pandemic, and most deaths occurred after 2 weeks of hospitalization.

## 1. Introduction

The coronavirus disease 2019 (COVID-19), which was first reported as a pneumonia of unknown etiology in Wuhan, Hubei Province, China, at the end of 2019, is an emerging viral illness caused by the novel beta coronavirus known as severe acute respiratory syndrome coronavirus-2 (SARS-CoV-2) [1, 2]. COVID-19 is an important global public health concern and is currently the leading cause of death from a single infectious agent, with an estimated 530 million cases resulting in over 6.3 million deaths in about 30 months [3].

COVID-19 is a multisystemic disease with varying clinical manifestations and severity depending on several host factors such as age, immune status, and the presence of co-morbidities [4, 5]. However, recently, some variants of SARS CoV-2 have been shown to be more transmissible and pathogenic and are associated with more symptomatic disease, severe/critical illness, and breakthrough infections in patients who have received the COVID-19 vaccine [6]. Most patients with COVID-19 are asymptomatic [7]. In those who are symptomatic; cough, chest pain, loss of smell, and nasal congestion as well as other extrapulmonary symptoms such as fever, joint paints, and headaches are the most common symptoms [8, 9]. However, about 5 to 15% of patients present with severe and/or critical disease characterized by acute respiratory distress syndrome and/or coagulopathy due to an exaggerated host immune response, also known as, the cytokine storm [10]. Previous studies have identified risk factors for severe/critical COVID-19 illness as the presence of comorbidities such as diabetes mellitus, hypertension, and obesity [11–13]. These are the group of individuals with the highest risk of mortality and morbidity due to COVID-19 [14].

Uganda has experienced 3 waves of the COVID-19 pandemic and recently experienced the third wave, with a rapid increase in the number of cases in mid-December 2021 and confirmation of the omicron variant, which spread very rapidly, globally. In the first wave, the first case of COVID-19 was reported on the 20^th^ of March 2020, and in the middle of April 2021, a second surge in the number of COVID-19 cases was observed. As of 27^th^ May 2022, over 164,000 cases and 3,596 COVID-19-related deaths have been reported as the country battles the third wave of the pandemic, predominantly with the omicron variant [3]. The first 100 cases were predominantly asymptomatic or mild cases with 100% survival [15, 16]. However, in the second wave, mortality was as high as 36% at the National COVID-19 Treatment Unit (CTU) [17].

Our understanding of the clinical presentation, radiological features, and outcomes of patients hospitalized with COVID-19 in Uganda is incompletely understood, with currently published studies based only on data from selected CTUs in public health facilities [16, 17]. However, data regarding the clinical presentation, imaging findings, and outcomes of patients with COVID-19 in private facilities in Uganda is still lacking. Here, we report on the clinical characteristics, treatment, and outcomes of patients with COVID-19 hospitalized at a CTU of a major private facility in Uganda.

## 2. Methods

### 2.1. Study Design

This was a descriptive, single-center, retrospective cohort study of patients hospitalized with COVID-19 at Case Hospital, Kampala, Uganda, between June 2020 and September 2021.

### 2.2. Study Setting

Case Hospital (also known as Case Medical Centre), established in 1995, is an urban, private, upscale, tertiary hospital situated in the heart of Kampala, Uganda. Case Hospital offers specialized, multidisciplinary healthcare in an 80-bed facility with a fully equipped intensive care unit, laboratory facilities, and state-of-the-art imaging modalities. Case Hospital started receiving patients with COVID-19 in early May 2020. The hospital has been accredited by the Ministry of Health of Uganda as a CTU.

### 2.3. Study Population

We included data of all patients hospitalized with a confirmed diagnosis of COVID-19 during the study period. COVID-19 was diagnosed by polymerase chain reaction and SARS-CoV-2 rapid antigen test, with or without a compatible chest imaging. We imaging. We excluded patients transferred out of the facility for whom outcome records could not be traced.

### 2.4. Data Collection

Three trained research assistants collected data regarding demographics (age, sex, occupation, level of education, etc.), comorbidities, HIV status, clinical symptoms and duration, clinical findings, severity of the disease, radiological findings, laboratory findings, prescription information concerning drugs (dose and duration), interventions (oxygen and mechanical ventilation), duration of hospitalization, and outcomes at discharge (alive or dead).

### 2.5. Definition of COVID-19 Disease Severity

Disease severity was defined according to the World Health Organization (WHO) Severity definitions [18].

#### 2.5.1. Critical COVID-19

Defined by the criteria for acute respiratory distress syndrome (ARDS), sepsis, septic shock, or other conditions that would normally require the provision of life-sustaining therapies such as mechanical ventilation (invasive or noninvasive) or vasopressor therapy.

#### 2.5.2. Severe COVID-19

Defined by any of (1) oxygen saturation <90% on room air, (2) respiratory rate >30 breaths/min in adults, (3) signs of severe respiratory distress (accessory muscle use, inability to complete full sentences, and, in children, very severe chest wall indrawing, grunting, central cyanosis, or the presence of any other general danger signs).

#### 2.5.3. Non-Severe COVID-19

Defined as the absence of any criteria for severe or critical COVID-19.

### 2.6. Data Analysis

Anonymized data was analyzed using STATA version 16.0 (StataCorp LLC, College Station, Texas, USA). We summarized continuous variables using means with standard deviations or medians with interquartile range (IQR) and categorical variables using frequencies and percentages. Chi-square test was performed to compare categorical variables, and Mann-Whitney U and *t*-tests for nonparametric and parametric continuous variables, respectively. A multivariable logistic regression model was constructed for the analysis of treatment outcomes accounting for all important confounders. The results of the logistic regression models were presented as odds ratios (ORs) with 95% confidence intervals (95% CIs). Kaplan–Meier survival analysis was performed to determine 14- and 28-day in-hospital mortality and time-to-event. For all analyses, *p* < 0.05 was considered statistically significant.

### 2.7. Ethical Considerations

The Mulago Hospital Research and Ethics Committee (MHREC) approved the study protocol and provided a waiver of informed consent of the study participants (approval reference: MHREC 2065). All ethical principles outlined in the *Declaration of Helsinki* were observed.

## 3. Results

### 3.1. Baseline Characteristics

**Figure 1:**
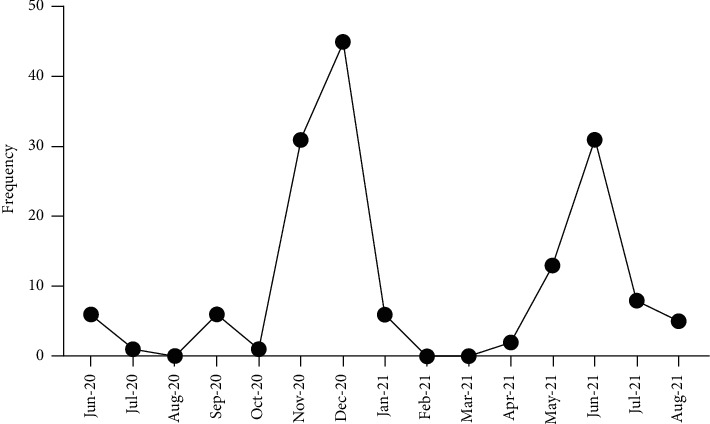
Trends in admission of patients with COVID-19 at case hospital.

Data of a total of 160 participants was eligible for analysis (Table 1). The median age was 45 years (IQR: 37–57) and 61 (38.9%) participants were 50 years of age or older. Sixty-seven (41.9%) participants had at least one comorbidity, with the majority having hypertension (26.9%, *n* = 43) or diabetes mellitus (16.9%, *n* = 27). There were surges in admissions between October–December 2020 and April–July 2021, coinciding with the first and second waves of COVID-19 in Uganda (Figure 1).

### 3.2. Clinical Presentation

**Figure 2:**
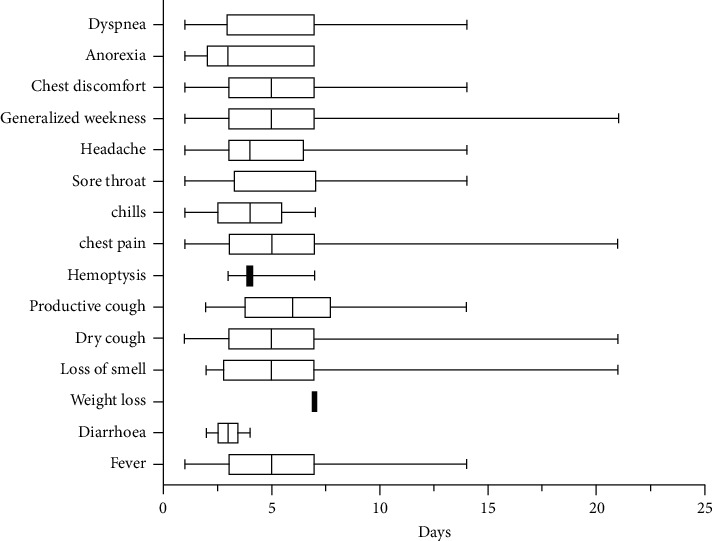
Duration of symptoms among COVID-19 patients at baseline.

Dry cough and general body weakness were the most frequent presenting complaints, present in 115 (71.9%) participants each (Table 1). More than half of the participants also had dyspnea (63.8%, *n* = 102) and fever (58.8%, *n* = 94). Eighteen (11.3%) participants had loss of smell. Three (1.9%) participants had hemoptysis and 5 (3.1%) had diarrhea. The median durations for all symptoms at admission (Figure 2) were ≤7 days, highest for sore throat (7 days, IQR: 3–7) and productive cough (6 days, IQR: 4–8), and the least for dyspnea (3 days, IQR: 2–7) and anorexia (3 days, IQR: 3–7).

### 3.3. Vital Status at Admission

**Figure 3:**
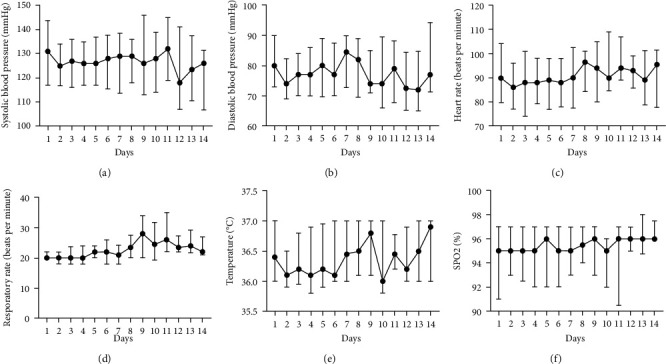
Vital status of the patients during the first two weeks of admission.

Table 1 also summarizes the vital measurements at admission. The median systolic and diastolic blood pressure was 131/80 mmHg. About 9 (5.6%) participants had hypotension at the time of admission. Forty-eight (30.4%) participants had tachycardia (heart rate ≥100) and only 4 (2.5%) had bradycardia (heart rate <60 beats per minute). Some 39 (41.1%) participants had tachypnoea, and none had bradypnea. Up to 25.9% (*n* = 41) of the participant had hypoxia (SpO_2_ <92%). Median vital measurements remained within normal limits over two weeks of monitoring (Figure 3).

### 3.4. Chest Imaging

**Figure 4:**
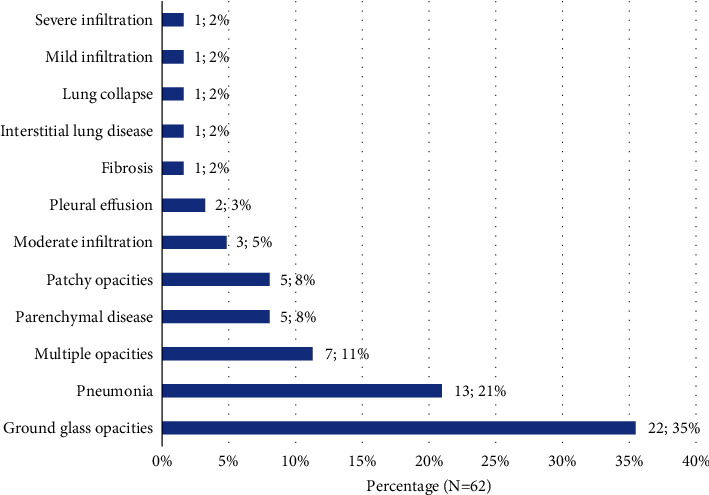
Chest CT findings among patients admitted with COVID-19 at case hospital, Uganda (*n* = 71). Data label presented as frequency; percentage.

Chest CT and/or X-ray scans were done in 88 (55%) patients and 77 (87.5%) had abnormal findings. The pathologies were reported in 62 chest CT findings, bilateral in 87% (*n* = 54) and unilateral in 8% (*n* = 5) participants.  Figure 4 shows the chest CT abnormalities recorded in the patient's files. More than one-third (35%, *n* = 22) noted ground-glass opacities, whereas 21% (*n* = 13) had findings suggestive of pneumonia. Patchy lung opacities were seen in 8% (*n* = 5) of the participants' chest CT scans. Bilateral lung fibrosis and interstitial lung disease were observed in one patient each (1.4%). Chest X-ray scans were done in four (2.5%) participants and were abnormal in three participants, which showed interstitial lung disease with fibrosis, basal pneumonia with fibrosis, and atypical pneumonia, each.

### 3.5. Laboratory Investigations

The median white blood cell count at admission was 7.2 × 10^6^ cells/mm^3^ with 34 (21.3%) and 17 (10.6%) having leukocytosis and leukopenia, respectively. Twenty-three (14.4%) participants had thrombocytopenia and 25 (15.6%) had anemia. The median values for renal function tests, electrolytes, and liver function tests were otherwise within normal ranges (Table 2).

### 3.6. COVID-19 Severity

**Figure 5:**
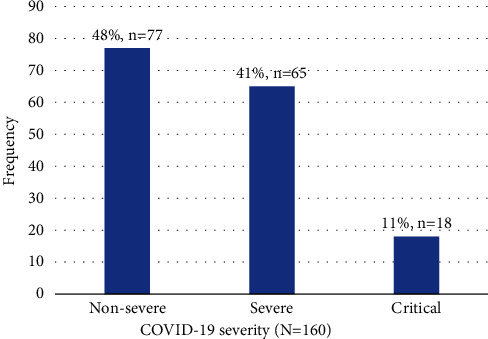
COVID-19 severity among the participants.

Seventy-seven (48.1%) participants had non-severe disease, whereas 18 (11.3%) had critical disease (Figure 5). More than half were categorized as severe critical COVID-19 disease (51.9%, *n* = 83).

### 3.7. Treatment and Patient Outcomes

Majority of the participants received azithromycin (76.9%), dexamethasone (75.0%), vitamin C supplementation (71.3%), vitamin D supplementation (71.3%), and enoxaparin (70.6%). Only 13 patients (8.1%) received remdesivir, whereas hydroxychloroquine was not given to any patient (Table 3). The dose of azithromycin given to patients was 500 mg once a day. The median duration on treatment was 3 days (interquartile range: 2–5 days). For dexamethasone, 41.2% received 4 mg and 39.5% received 8 mg daily, with a median duration of 3 (IQR: 2.0–4.5) days. About 50% of the patients on enoxaparin were treated with 40 mg daily, 45.5% received 60 mg daily, and 4.5% received 80 mg daily.

**Figure 6:**
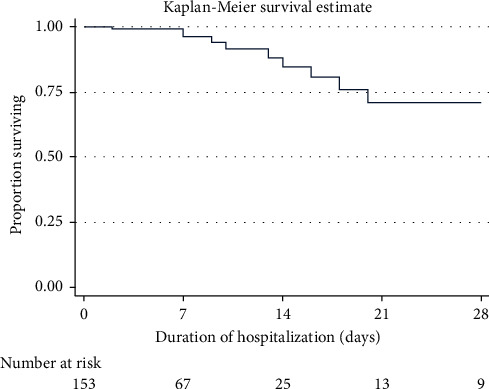
Survival among the participants.

Overall, a total of 18 patients (11.3%) with COVID-19 disease died during the study period. Survival was 99% at 7 days, 89% at 14 days, and 72% at both 21 days and 28 days (Figure 6). At bivariate analysis (Table 4), participants who died were significantly older than those who survived (median age: 58 (IQR: 55–61) *versus* 42 (IQR: 36–56) years, *p* < 0.001). Of the 18 participants who died, 14 (78%) were older than 50 years of age. Stratified age was also significantly associated with death (*p*=0.005). Participants with at least one comorbidity also significantly had a high mortality compared to those who did not (21.8% *versus* 6.9%, *p*=0.024). Having hypertension (*p*=0.026) and diabetes (*p*=0.048) were significantly associated with mortality. However, mortality did not differ by gender (*p*=0.805). Participants who died significantly had a higher respiratory rate (24 (20–36) *versus* 20 (20–22), *p*=0.002) and a lower SpO_2_ (87 (75–98) v*ersus* 95 (92–97), *p*=0.031 at admission compared to those who survived. A significantly smaller proportion of participants who received ivermectin (*p*=0.002) and remdesivir died (*p*=0.042).

### 3.8. Factors Associated with Mortality

Table 5 shows the factors that were associated with mortality in patients with COVID-19 in this study. Participants aged 50 years and above were 8.6 times (OR: 8.6, 95% CI: 1.1–69.2, *p*=0.042) more likely to die compared to their younger counterparts (18–35 years). Similarly, patients with at least one comorbidity were three times more likely to die (OR: 3.2, 95% CI: 1.1–8.9, *p*=0.029). Participants with hypertension (OR: 3.2, 95% CI: 1.2–8.6, *p*=0.024) and diabetes mellitus (OR: 2.9, 95% CI: 1.0–8.5, *p*=0.056) were more likely to die than their counterparts. An increase in respiratory rate was associated with a 10% increase in the likelihood to die (OR: 1.1, 95% CI: 1.0–1.2, *p*=0.002). A SpO_2_ <92% was associated with 5-fold high odds of dying (OR: 5.1, 95% CI: 1.8–14.4, *p*=0.002). Patients treated with either ivermectin or remdesivir were 80% less likely to die (Table 5).

## 4. Discussion

In the beginning of the COVID-19 pandemic in Uganda, all patients diagnosed with COVID-19 were hospitalized only in major public health facilities. Private health facilities were only cleared to manage patients with COVID-19 when the number of cases had significantly increased, overwhelming the public health facilities. This is the first study to describe the clinical outcomes of patients with COVID-19 hospitalized in a private facility in Uganda. In this study, we report several important findings.

First, over half of the patients in our study presented with severe or critical illness, with about 11% of the patients requiring ICU admission and invasive mechanical ventilation. In the study by Bongomin and colleagues at Mulago National Referral Hospital (MNRH) CTU, a public facility and the largest CTU in Uganda, over 80% of patients presented with severe/critical COVID-19 illness, with up to 20% of patients requiring ICU admissions [17]. The observed difference could be because the majority of patients admitted to MNRH CTU are referral cases from across the country. These findings agree with the published data showing a high proportion of patients presenting with severe or critical COVID-19 illness during the second wave attributed to the emergence of delta and other variants of the SARS CoV-2 [19, 20].

Chest radiographs may be normal in early or mild disease. We noted that about 12% of our participants had normal chest CT images. However, of the 80% of the patients who had abnormal chest imaging findings, over 90% had bilateral disease. Chest imaging is a key in the diagnosis of COVID-19, grading its severity, as well as the investigation of complications such as acute respiratory distress syndrome (ARDS), embolism, and pulmonary infections [21, 22]. Although chest CT may be more sensitive than chest radiograph and some chest CT findings may be characteristic of COVID-19, no finding can completely rule in or rule out the possibility of COVID-19. In the United States, the American College of Radiology (ACR) does not recommend using chest CT for screening or diagnosing of COVID-19 and recommends reserving it for hospitalized patients when needed for management. Chest CT in patients with COVID-19 most commonly demonstrates ground-glass opacification with or without consolidative abnormalities, consistent with viral pneumonia [22–24].

Secondly, we found an overall in-hospital mortality of approximately 11%, with a significantly higher mortality of 26% during the second wave compared to 5% during the first wave of the COVID-19 pandemic in Uganda. Our findings are consistent with previous reports from Uganda showing a higher mortality during the second wave of the COVID-19 pandemic in Uganda [16, 17]. However, the mortality reported in this study is much lower than that reported at MNRH CTU [17]. A higher proportion of patients presenting to MNRH CTU had severe/critical illness compared to those in our setting (>80% versus 52%), and challenges related to late presentation and shortage of oxygen and healthcare workers were also reported to have contributed to the high mortality in public health facilities across Uganda. Mortality reported in our study is also much lower than the 32% reported in Cameroon [25], and 48·2% in a cohort study of over 3,000 critically ill patients with COVID-19 pneumonia enrolled in 64 hospitals in ten African countries [26]. In the latter study, in addition to the traditional risk factors for adverse COVID-19 outcome, persons living with HIV/AIDS and those who experienced delayed access to high-care units and ICU had higher mortality rates.

Thirdly, we showed that advanced age, diabetes mellitus, hypertension, and hypoxemia were significantly associated with mortality. These findings are consistent with studies from Uganda [17] and several meta-analyses which showed increased severity and mortality in patients with co-morbidities [27, 28]. The relationship between COVID-19 and comorbidities such as diabetes mellitus and hypertension are bilateral. With COVID-19 increasing the incidence and exacerbating the control of these conditions, and yet these conditions also worsen COVID-19 outcomes [4, 29, 30].

There are currently several drugs recommended by the WHO for the treatment of COVID-19 [31]. These include the Pfizer combination nirmatrelvir and ritonavir, molnupiravir, sotrovimab, and remdesivir for non-severe disease [31]. Molnupiravir is an oral antiviral agent with clinical utility in the treatment of mild moderate disease, shortening the duration of hospitalization, and halting disease progression to severe or critical illness [31]. Corticosteroids like dexamethasone, tocilizumab (IL-6 receptor blocker), and baricitinib (JAK 1 and JAK 2 inhibitor) are indicated in the management of patients with severe to critical COVID-19 [31]. In this study, we found that remdesivir and ivermectin were each associated with an 80% reduction in mortality. Previous studies have shown that remdesivir may be used as an add-on therapy for patients with severe/critical illness [32, 33]. However, recent studies have failed to show any benefit of ivermectin for the treatment of both moderate and severe/critical illness [34].

It is also important to note that only a small proportion of patients in our facility received ivermectin and remdesivir, and the wide confidence interval indicates a lack of precision. Therefore, these positive findings should be cautiously interpreted. The massive use of azithromycin and vitamins in our study is attributed to guidance from the first edition of Uganda national guidelines for COVID-19 management recommending their use even for mild-moderate disease [35]. The frequent use of ulinastatin in our study could be supported by studies that reported some studies that reported some benefits, especially for COVID-19 patients with sepsis, or moderate-severe disease [36, 37]. Our agreement is that clinicians should follow updated guidelines by their respective health authorities/organization while accounting for the evolving data on the safety and efficacy of various therapeutic agents.

Our study has some important limitations. First, it was a retrospective review of medical records and not all relevant data could be obtained for all participants. For example, no data was extracted on COVID-19 vaccination status of the participants. Secondly, this was a single-center study, recruiting mainly participants from higher socioeconomic status around the capital city, and may not be generalized to other private facilities, particularly in upcountry settings. Despite this, we had a robust dataset including clinical, laboratory, and radiological variables, which provides a better understanding of the complete picture of COVID-19 manifestation in Ugandans. Our findings inform clinical practice and future studies to optimize the clinical outcomes of patients with COVID-19 in Uganda and similar settings.

## 5. Conclusions

In conclusion, in a private clinical practice where about half of the patients presented with severe or critical COVID-19 illness, about 1 in 10 died, with a significantly higher proportion of patients dying in the second wave compared to the first wave of the COVID-19 pandemic in Uganda. Most deaths, however, occurred after 7 days of hospitalization.

## Figures and Tables

**Table 1:** Demographic and clinical characteristics at admission among participants with confirmed COVID-19 at case hospital.

Demographics	Frequency/median	Percentage/interquertile range
Age: (median, interquertile range) years	45	37–57
Age category		
18–35	30	19.1
36–40	62	39.5
<18	4	2.6
≥50	61	38.9

Sex (*n* = 159)		
Female	58	36.5
Male	101	63.5

Occupation		
Formal	10	6.3
Informal	2	1.3
Unknown	148	92.5

Comorbidities		
None	93	58.1
At least one comorbidity	67	41.9

Types of comorbidities		
Hypertension	43	26.9
Diabetes	27	16.9
HIV	3	1.9
Others	13	8.1

Symptoms at admission		
Dry cough	115	71.9
General body weakness	115	71.9
Dyspnoea	102	63.8
Fever	94	58.8
Chest pain	71	44.4
Chest discomfort	41	25.6
Sore throat	24	15
Headache	24	15
Loss of smell	18	11.3
Productive cough	18	11.3
Anorexia	7	4.4
Others^*∗*^	7	4.4
Chills	6	3.8
Diarrhea	5	3.1
Haemoptysis	3	1.9
Weight loss	1	0.6

*Vital measurements*	*Median*	*Interquartile range*
Systolic blood pressure (mmHg, *n* = 156)	131	117–144
Diastolic blood pressure (mmHg, *n* = 156)	80	73–90
Heart rate (beats per minute, *n* = 158)^*∗∗*^	90	80–104
Respiratory rate (breaths per minute, *n* = 95)^*∗∗∗*^	20	20–22
Temperature (°C, *n* = 151)^*∗∗∗∗*^	36	36–37
Random blood sugar (mmol/L, *n* = 40)^*∗∗∗∗∗*^	9	6–14
SPO_2_ (%, *n* = 158)^*∗∗∗∗∗∗*^	95	91–97
Length of hospitalization (days)	5	3–9

^*∗*^Other symptoms included vomiting, lower back pain, joint pains, convulsion, abdominal swelling, left-sided weakness, and altered mental state, in one patient each. ^*∗∗*^Tachycardia (≥100 beats per minute) was present in 48 (30.4%) patients. ^*∗∗∗*^Tachypnea (>20 breaths per minute) was present in 39 (41.1%) patients. ^*∗∗∗∗*^Fever (≥38.0°C) was present in 11 (7.3%) patients. ^*∗∗∗∗∗*^Hyperglycemia (≥11.1 mmol/L) was present in 18 (45%) patients. ^*∗∗∗∗∗∗*^Hypoxia (<92%) was present in 41 (25.9%).

**Table 2:** Median laboratory parameters among COVID-19 patients admitted at case hospital.

Laboratory variables	*n*	Median	Interquartile range
Complete blood count			
White blood cells	150	7.2	5.4–10.6
Basophils	149	0.02	0.01–0.02
Lymphocytes	151	1.2	0.8–1.9
Eosinophils	148	0.01	0.0–0.06
Monocytes	149	0.5	0.3–0.7
Platelets	152	215	176–276
Haemoglobin	149	14.4	12.7–15.9
MCV	149	85.2	81.1–89.5

Renal function test			
Urea	129	4.1	3.1–6.1
Creatinine	137	75.7	61.7–96.1
Sodium	139	138	134–140
Potassium	138	4.1	3.7–6.4
Magnesium	16	0.8	0.7–1.1
Calcium	14	1.9	1.8–2.1
Chloride	138	102	99–110

Liver function test			
ALT	127	34	23–59
AST	121	35	24–60
ALP	63	63	50–87
GGT	122	56	33–111
Albumin	131	38.3	33.6–41.5
International normalized ratio	37	1.35	1.16–1.66
Total bilirubin	129	10.3	7.4–14.4
Direct bilirubin	121	5.1	3.5–7.0
Vitamin D	18	24.6	16.1–138.4

**Table 3:** Treatment modalities administered to patients admitted at case hospital.

Medication (*N* = 160)	Received: frequency (%)	Modal dose (%)	Duration (median days, IQR)
Azithromycin	123 (76.9)	500 mg	3 (2–5)
Dexamethasone	120 (75)	4 mg (41.2%)	3 (2–4.5)
Vitamin C	114 (71.3)	500 mg	4 (2–5)
Vitamin D	114 (71.3)	1000 IU	4 (2–5)
Enoxaparin	113 (70.6)	40 mg (50%)	3 (2–5)
Zinc	106 (66.3)	20 mg	3 (2–5)
Ulinastatin	96 (60)	100000 IU (60.2%)	3 (2–5)
Ivermectin	18 (11.3)	12 mg	3.5 (2–5)
Remdesivir	13 (8.1)	100 mg	4 (2–4)
Warfarin	1 (0.6)	2.5 mg	2 (NA)

**Table 4:** Distribution of mortality across patient demographics, admission vitals and medications administered to patients with COVID-19 at case hospital.

Demographics	Died: *n* (%) *N* = 18	Alive: *n* (%) *N* = 142	*p*-value
Age in years	58 (55–61)	42 (36–56)	0.001

Age categories			
18–35	1 (3.3)	29 (96.7)	0.005
36–40	3 (4.6)	62 (95.4)	
<18	14 (23)	47 (77)	
≥50	0 (0)	4 (100)	

Sex			
Female	7 (12.1)	51 (87.9)	0.805
Male	11 (10.8)	91 (89.2)	

Occupation			
Formal	2 (20)	8 (80)	0.470
Informal	0 (0)	2 (100)	
Unknown	16 (11)	130 (89)	

Comorbidities			
None	6 (6.5)	87 (93.5)	0.024
At least one	12 (17.9)	55 (82.1)	

Hypertension			
No	9 (7.7)	108 (92.3)	0.026
Yes	9 (20.9)	34 (79.1)	

Diabetes mellitus			
No	12 (9)	121 (91)	0.048
Yes	6 (22.2)	21 (77.8)	

HIV			
No	17 (10.8)	140 (89.2)	0.303
Yes	1 (33.3)	2 (66.7)	

Others			
No	16 (10.9)	131 (89.1)	0.643
Yes	2 (15.4)	11 (84.6)	

Symptoms at admission			
Asymptomatic	1 (33.3)	2 (66.7)	0.303
Symptomatic	17 (10.8)	140 (89.2)	

Vitals at admission			
Systolic blood pressure	131 (116–154)	131 (117–141)	0.772
Diastolic blood pressure	76 (64–82)	80 (73–90)	0.151
Heart rate	96 (83–124)	89 (80–102)	0.253
Respiratory rate	24 (20–36)	20 (20–22)	0.002
Temperature	36.4 (35.9–37.0)	36.4 (35.4–36.9)	0.852
Glasgow comma scale	15 (15–15)	15 (15–15)	>0.999
Random blood sugar	10.5 (4.7–16.3)	8.5 (5.8–14.1)	0.955
SpO_2_	87 (75–98)	95 (92–97)	0.031
≥92%	7 (6.0)	110 (94.0)	0.001
<92%	10 (24.4)	31 (75.6)	

Medications			
Azithromycin	14 (11.4)	109 (88.6)	>0.999
Dexamethasone	13 (10.8)	107 (89.2)	0.773
Vitamin C	14 (12.3)	100 (87.7)	0.593
Vitamin D	14 (12.3)	100 (87.7)	0.593
Enoxaparin	15 (13.3)	98 (86.7)	0.277
Zinc	12 (11.3)	94 (88.7)	0.968
Ulinastatin	11 (11.5)	85 (88.5)	0.919
Ivermectin	6 (33.3)	12 (66.7)	0.002
Remdesivir	4 (30.8)	9 (69.2)	0.042
Warfarin	0 (0)	1 (100)	>0.999

**Table 5:** Factors associated with mortality due to COVID-19 among the study participants.

Variables	Odds ratio (95% CI)	*p*
Age in years		
18–35	1.0	
36–40	1.4 (0.1–14.1)	0.773
≥50	8.6 (1.1–69.2)	0.042

Sex		
Female	1.0	
Male	0.9 (0.3–2.4)	0.805

Occupation		
Formal	1.0	
Unknown	0.5 (0.1–2.5)	0.395

Comorbidities		
None	1.0	
At least one	3.2 (1.1–8.9)	0.029

Hypertension		
No	1.0	
Yes	3.2 (1.2–8.6)	0.024

Diabetes mellitus		
No	1.0	
Yes	2.9 (1.0–8.5)	0.056

HIV		
No	1.0	
Yes	4.1 (0.4–47.8)	0.258

Others		
No	1.0	
Yes	1.5 (0.3–7.3)	0.625

Symptoms at admission		
Asymptomatic	1.0	
Symptomatic	0.2 (0–2.8)	0.258

Vitals at admission		
Systolic blood pressure	1.0 (1.0–1.0)	0.879
Diastolic blood pressure	1.0 (0.9–1.0)	0.123
Heart rate	1.0 (1.0–1.0)	0.207
Respiratory rate	1.1 (1.0–1.2)	0.002
Temperature	1.2 (0.7–2.2)	0.511
Random blood sugar	1.0 (0.8–1.2)	0.945
SpO_2_ <92	5.1 (1.8–14.4)	0.002

Medications		
Azithromycin	0.9 (0.3–3.1)	0.923
Dexamethasone	1.2 (0.4–3.5)	0.773
Vitamin C	0.7 (0.2–2.2)	0.518
Vitamin D	0.7 (0.2–2.2)	0.518
Enoxaparin	0.4 (0.1–1.6)	0.219
Zinc	1.0 (0.3–2.8)	0.968
Ulinastatin	0.9 (0.3–2.6)	0.919
Ivermectin	0.2 (0.1–0.6)	0.004
Remdesivir	0.2 (0.1–0.9)	0.030

## Data Availability

The data that support the findings of this study is available on request from the corresponding author and approval from the administration of Case Hospital, Uganda.
